# Effects of Olympic Combat Sports on Health-Related Quality of Life in Middle-Aged and Older People: A Systematic Review

**DOI:** 10.3389/fpsyg.2021.797537

**Published:** 2022-01-05

**Authors:** Pablo Valdés-Badilla, Tomás Herrera-Valenzuela, Eduardo Guzmán-Muñoz, Pedro Delgado-Floody, Cristian Núñez-Espinosa, Matias Monsalves-Álvarez, David Cristóbal Andrade

**Affiliations:** ^1^Department of Physical Activity Sciences, Faculty of Education Sciences, Universidad Católica del Maule, Talca, Chile; ^2^Carrera de Entrenador Deportivo, Escuela de Educación, Universidad Viña del Mar, Viña del Mar, Chile; ^3^Department of Physical Activity, Sports and Health Sciences, Faculty of Medical Sciences, Universidad de Santiago de Chile, USACH, Santiago, Chile; ^4^Escuela de Kinesiología, Facultad de Salud, Universidad Santo Tomás, Talca, Chile; ^5^Department of Physical Education, Sports and Recreation, Universidad de La Frontera, Temuco, Chile; ^6^Centro Asistencial Docente y de Investigación, Universidad de Magallanes, Punta Arenas, Chile; ^7^School of Medicine, Universidad de Magallanes, Punta Arenas, Chile; ^8^Instituto de Ciencias de la Salud, Universidad de O'Higgins, Rancagua, Chile; ^9^Human Performance Laboratory, Motion Training, Rehab & Nutrition, Lo Barnechea, Chile; ^10^Departamento Biomédico, Centro de Investigación en Fisiología y Medicina de Altura (MedAlt), Facultad de Ciencias de la Salud, Universidad de Antofagasta, Antofagasta, Chile

**Keywords:** martial arts, exercise, mental health, health promotion, ageing

## Abstract

Olympic combat sports are unconventional physical activity strategies to train middle-aged and older people with and without health problems. This systematic review aimed to assess the available body of published peer-reviewed articles related to the effects of Olympic combat sports interventions (boxing, fencing, judo, karate, taekwondo, wrestling) on health-related quality of life in adults aged 45 and older. The search was carried out in five generic databases until July 2021 and the protocol was registered in PROSPERO (code: CRD42021244161). The PRISMA guidelines were followed and the Downs and Black checklist was used to assessed the methodological quality of the studies. After reviewing 1,151 records, only seven studies met the inclusion criteria, adding 212 participants (43.4% female) with a mean age of 63.7 years. Six studies (two with middle-aged participants and four with older people) provided data to calculate the effect size (ES) in the Olympic combat sports groups (No research that used taekwondo or wrestling as an intervention modality was found). Three studies reported beneficial changes with a small ES for the total score (*d* < 0.40) of the health-related quality of life. Two studies reported a beneficial change with a small ES (*d* = 0.49) and strong ES (*d* = 4.45) for physical health. One study reported improvements with a small ES for emotional (*d* = 0.23) and functional (*d* = 0.26) well-being. In conclusion, interventions based on Olympic combat sports produce beneficial effects with a small and moderate ES on health-related quality of life in male and female aged 45 and older who are healthy participants, participants with Parkinson's disease, and participants with breast cancer.

**Systematic Review Registration:** https://www.crd.york.ac.uk/prospero/, PROSPERO: CRD42021244161.

## Introduction

Physical activity (PA) is considered an essential element to achieve healthy ageing (Fragala et al., 2019; Bull et al., 2020) and distinguished as the cheapest alternative for disease prevention (Reis et al., 2016). Various systematic reviews with and without meta-analysis have confirmed the benefits of regular PA practise on different health variables in middle-aged and older people (Warburton and Bredin, 2017; Zubala et al., 2017; Valdés-Badilla et al., 2019; Grande et al., 2020). Contrarily, physical inactivity increases the risk of suffering cardiovascular and all-cause mortality (Cunningham et al., 2020). Further, physical inactivity is related to cognitive decline, dementia, depression, and even Alzheimer's disease (Cunningham et al., 2020), facts that as a whole have a negative impact on the health-related quality of life (HRQoL) in older people (Kojima et al., 2016; Ingrand et al., 2018).

Classically, PA strategies used to train adults and older people with and without health problems correspond to interventions based on resistance (Grgic et al., 2018; Fragala et al., 2019) and multi-component training (Cadore et al., 2013; Vargas-Vitoria et al., 2021), possibly due to greater dissemination, scientific support, and versatility to adapt the workload and exercises to the participants' characteristics (Fragala et al., 2019). These interventions have achieved significant improvements in HRQoL in healthy people (Marquez et al., 2020), and patients with heart failure (Giuliano et al., 2017) and cancer (Mishra et al., 2012). Consideing the beneficial effects of PA on health outcome, but at the same time, showing the great diversity of interventions that exist today, it is necessary to clarified the effects, but not only of the classical intervention, if not in strategies based on unconventional disciplines.

Among PA strategies based on unconventional disciplines, the martial arts and combat sports have also shown significant improvements in HRQoL in healthy older people who practise kendo (Mendonça et al., 2017), as well as in people with Parkinson's disease treated with tai-chi (Song et al., 2017) and cancer patients undergoing kyusho jitsu sessions (Strunk et al., 2018). Although martial arts and combat sports have shown health benefits in different population groups (Bu et al., 2010; Origua Rios et al., 2018; Moore et al., 2020), interventions with Olympic combat sports (OCS) are less known, probably for being considered risky activities (Bromley et al., 2018). However, a recent systematic review reported improvements in physical-functional, physiological, and psychoemotional health in older people (Valdés-Badilla et al., 2021), but so far, no specific work has been published that synthesises the effects of OCS on HRQoL in middle-aged and older people. In this aspect, a systematic review could contribute new insights for healthcare and sports science professionals that can help them to make better-informed, evidence-based decisions regarding use from OCS. Therefore, the present systematic review aimed to assess the available body of published peer-reviewed articles related to the effects of OCS interventions (boxing, fencing, judo, karate, taekwondo, wrestling) on HRQoL in adults aged 45 and older.

## Methods

This systematic review followed the PRISMA (Preferred Reporting Items for Systematic Reviews and Meta-analyses Protocols) guidelines (Page et al., 2021). It was also registered on PROSPERO (International Prospective Register of Systematic Reviews; code: CRD42021244161). And it followed the methodological recommendations of a previous systematic review in OCS (Valdés-Badilla et al., 2021).

### Eligibility Criteria

The inclusion criteria for this review were the following: (i) original articles peer-reviewed without limitations on language or publication date, published until July 2021; (ii) a study population that is middle-aged and older people, regardless of gender, considering as middle-aged people mean aged 45 years or older (Livingston et al., 2020); (iii) interventions with OCS (boxing, fencing, judo, karate, taekwondo, wrestling) lasting 4 weeks or more; (iv) with or without a control group; (v) at least one HRQoL measurement (e.g., the Short Form Health Survey, Brief quality-of-life survey, the Euroquol-5D) pre-and post-intervention; and (vi) a study design that was experimental (randomised-controlled trials or non-randomised trials) or longitudinal with at least two assessments (pre-and post-intervention). The exclusion criteria were as follows: (i) studies with an intervention that did not focus on OCS; (ii) a study population that is under 45 years of age; (iii) studies without post-evaluation (iii) studies that were not original research publications (e.g., letters to the editor, translations, notes, book reviews), duplicate articles, review articles (e.g., meta-analyses, systematic reviews, narrative reviews) or case studies; (iv) cross-sectional, retrospective, and prospective studies.

### Information and Database Search Process

The information search was carried out between May and July 2021 in the following databases: SCOPUS, PubMed/MEDLINE, Web of Science (all databases), social and behavioural sciences PsycINFO (American Psychological Association), and the collection of Psychology and Behavioural Sciences (EBSCO). The Medical Subject Headings (MeSH) from the United States of America National Library of Medicine were used, and bias-free language terms related to HRQoL, OCS, and middle-aged and older people were used. The following search string was used: (“quality of life” OR “health related quality of life” OR “health-related quality of life” OR “mental health” OR “psychological health”) AND (“boxing” OR “fencing” OR “judo” OR “karate” OR “taekwondo” OR “wrestling”) AND (“adults” OR “middle aged” OR “middle-aged” OR “elderly” OR “older adults” OR “older people” OR “older subject” OR “ageing” OR “ageing” OR “aged”). To include the most recent studies in the review, quote alarms were set in the respective databases; thereby, the principal researcher automatically received emails regarding the latest updates of the search terms used. These updates were received daily (if they were available), and the studies were eligible for inclusion until the manuscript preparation (July 2021). After the formal systematic searches, additional manual searches were performed by consulting grey literature (e.g., conference proceedings), considered if the complete text was available. Besides, the included studies' reference lists were reviewed, and prior reviews and meta-analyses were examined to detect studies that were potentially eligible for inclusion.

### Study Selection and Data Collection Process

The studies were exported to the EndNote references manager (version X8.2, Clarivate Analytics, Philadelphia, PA, USA), where they were filtered once again by selecting the title, abstract, and keywords. In some cases, it was necessary to cheque the full text. Two authors (PVB, THV) independently conducted the selection and data collection processes. Discrepancies between the two authors regarding the study conditions were resolved through consensus with a third author (EGM). Afterwards, the full text of the potentially eligible studies was reviewed, and the exclusion reasons of those studies that did not meet the selection criteria were informed. The studies' data were extracted by two authors independently using a form created through Microsoft Excel (Microsoft Corporation, Redmond, WA, USA).

### Methodological Quality Assessment

The objective of this phase was to detect the risk of bias for each of the selected studies, which could eventually lead to the exclusion of some of the previously chosen studies. For this purpose, the Downs and Black (1998) checklist was applied, a tool that has been widely used in health care interventions. It is made up of 27 criteria (details in Supplementary Material), which are related to reporting (10 items), external validity (3 items), internal validity—bias (7 items), internal validity—confusion (selection bias; 6 items), and statistical power (1 item). All criteria have a value of 0 to 1 point, except for two criteria, one with a maximum score of 2 points (criterion no. 5) and one with a maximum score of 5 points (criterion no. 27), allowing a study to be scored between 0 and 32 points (Downs and Black, 1998). The complete list is usually applied for randomised-controlled trials. At the same time, for non-randomised studies, it is reduced to 17 criteria, after excluding criteria 9, 13, 14, 17, 19, 22, 23, 24, 26, and 27, which are not applicable in non-randomised studies, allowing such studies to be rated with a maximum score of 17 points (Downs and Black, 1998; Freke et al., 2016). This process was conducted by two authors (PVB, THV) independently from one another, and a third author (EGM) acted as a referee in doubtful cases, which were then validated by another author (PVB).

### Data Synthesis

The following data were obtained and analysed from the selected studies: (i) author and publication year; (ii) country of origin; (iii) study design; (iv) intial health of the sample; (v) groups: total number of participants, mean age, intervention groups, and gender; (vi) activities developed in the intervention; (vii) training volume (total duration, weekly frequency, and time per session); (viii) training intensity; (ix) measurement of HRQoL; (x) main outcomes of the studies; and (xi) analysis of the systematic review.

### Data Analysis

Meta-analyses were included in the study protocol, with full details available at PROSPERO, registry code CRD42021244161. However, the diversity of instruments used to measure HRQoL and the small number of randomised-controlled trials precluded a robust meta-analysis. Nevertheless, complementary analyses were carried out using the means and standard deviations (SDs) before and after the intervention of the total score, settings, or health dimensions of the HRQoL for the studies that provided data from the OCS groups interventions. The effect size (ES) was calculated with Cohen's *d* (Cohen, 1992), considering a small (0.20–0.49), moderate (0.50–0.79), or strong effect (> 0.80). All analyses were carried out using the Comprehensive Meta-Analysis software (version 2; Biostat, Englewood, NJ, USA).

## Results

### Study Selection

**Figure 1:**
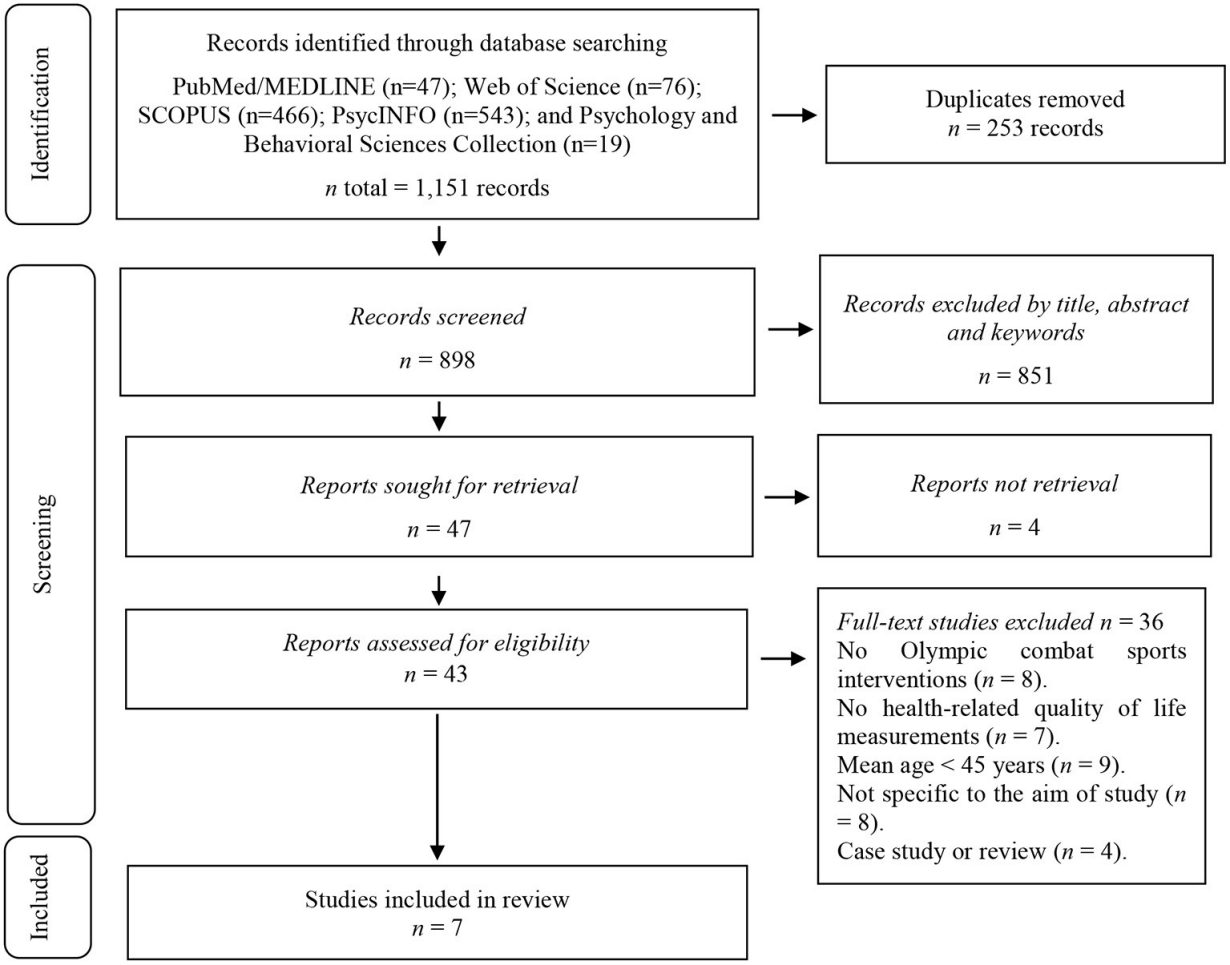
Flowchart of the review process^#^. ^#^Based on PRISMA guidelines (Page et al., 2021).

The search process is detailed in Figure 1. A total of 1,151 registers were found during the study identification stage. During the screening phase, duplicates were eliminated, and the studies were filtered by selecting the title, abstract, and keywords, thus obtaining 47 references. Four studies were excluded for being inaccessible, so the full texts of 43 studies were analysed. Eight studies were excluded because they were not OCS interventions; seven for not having HRQoL measurements; nine because the mean age of the sample was <45 years of age; eight because they did not correspond to the research object (i.e., not centred on the HRQoL outcomes of adults 45 years and older); four for not being able to access the full texts of the studies; and four because they were reviews or case studies. After this process, seven studies met the selection criteria (Marie-Ludivine et al., 2010; Combs et al., 2013; Jansen et al., 2017; Lantheaume et al., 2017; Ciaccioni et al., 2019; Dawson et al., 2020; Fleisher et al., 2020).

### Methodological Quality

The selected studies were analysed with the Downs and Black (1998) checklist. All studies obtained 60% or more of the total scale score for randomised (32 points) and non-randomised (17 points) controlled trials, as shown in Table 1. Two studies obtained a score of 28/32 (Combs et al., 2013; Ciaccioni et al., 2019), one of 27/32 (Jansen et al., 2017), three of 14/17 (Marie-Ludivine et al., 2010; Dawson et al., 2020; Fleisher et al., 2020), and one of 11/17 (Lantheaume et al., 2017), indicating a moderate to high methodological quality, so no studies were excluded from the systematic review.

**Table 1:** Methodological quality assessment of studies^#^.

**Criteria**	**Ciaccioni et al. (2019)^**a**^**	**Combs et al. (2013)^**a**^**	**Dawson et al. (2020)^**b**^**	**Fleisher et al. (2020)^**b**^**	**Jansen et al. (2017)^**a**^**	**Lantheaume et al. (2017)^**b**^**	**Marie-Ludivine et al. (2010)^**b**^**
1	Yes	Yes	Yes	Yes	Yes	Yes	Yes
2	Yes	Yes	Yes	Yes	Yes	Yes	Yes
3	Yes	Yes	Yes	Yes	Yes	Yes	Yes
4	Yes	Yes	Yes	Yes	Yes	No	Yes
5^*^	Partially	Partially	No	No	Partially	No	No
6	Yes	Yes	Yes	Yes	Yes	Yes	Yes
7	Yes	Yes	Yes	Yes	Yes	Yes	Yes
8	Yes	Yes	Yes	Yes	Yes	Yes	Yes
9	Yes	Yes	Not applicable	Not applicable	Yes	Not applicable	Not applicable
10	Yes	Yes	Yes	Yes	Yes	Yes	Yes
11	No	No	No	No	No	No	No
12	Yes	Yes	Yes	Yes	Yes	No	Yes
13	Yes	Yes	Not applicable	Not applicable	Yes	Not applicable	Not applicable
14	No	Yes	Not applicable	Not applicable	No	Not applicable	Not applicable
15	Yes	Yes	No	No	No	No	No
16	Yes	Yes	Yes	Yes	Yes	Yes	Yes
17	Yes	Yes	Not applicable	Not applicable	Yes	Not applicable	Not applicable
18	Yes	Yes	Yes	Yes	Yes	Yes	Yes
19	Yes	Yes	Not applicable	Not applicable	Yes	Not applicable	Not applicable
20	Yes	Yes	Yes	Yes	Yes	Yes	Yes
21	Yes	Yes	Yes	Yes	Yes	No	Yes
22	Yes	Yes	Not applicable	Not applicable	Yes	Not applicable	Not applicable
23	Yes	Yes	Not applicable	Not applicable	Yes	Not applicable	Not applicable
24	Unable to determine	Unable to determine	Not applicable	Not applicable	Unable to determine	Not applicable	Not applicable
25	Yes	Yes	Yes	Yes	Yes	Yes	Yes
26	Yes	Yes	Not applicable	Not applicable	Yes	Not applicable	Not applicable
27^**^	5	4	Not applicable	Not applicable	5	Not applicable	Not applicable
Total score	28	28	14	14	27	11	14

# *According to the Downs and Black (1998) checklist*.

* *The score goes from 0 to 2 points*.

** *The score goes from 0 to 5 points. Criteria: methodological quality criteria (details in Supplementary Material). Not applicable: 0. Partially: 1 point. Unable to determine: 0. Yes: 1 point*.

a *Randomised-controlled trial maximum score of 32 points*.

b *Non-randomised trial maximum score 17 points*.

### Study Characteristics

Regarding the type of study, three were randomised-controlled trials (Combs et al., 2013; Jansen et al., 2017; Ciaccioni et al., 2019), one was a non-randomised (pre-experimental) trial (Fleisher et al., 2020), and three had a longitudinal design (Marie-Ludivine et al., 2010; Lantheaume et al., 2017; Dawson et al., 2020). Of these, three studies were developed in the United States of America (Combs et al., 2013; Dawson et al., 2020; Fleisher et al., 2020), one in Canada (Marie-Ludivine et al., 2010), one in Italy (Ciaccioni et al., 2019), one in Germany (Jansen et al., 2017), and one in France (Lantheaume et al., 2017). Regarding the modality of OCS practised, three were interventions with karate (Marie-Ludivine et al., 2010; Jansen et al., 2017; Fleisher et al., 2020), two with boxing (Combs et al., 2013; Dawson et al., 2020), one with judo (Ciaccioni et al., 2019), and one with fencing (Lantheaume et al., 2017). No research that used taekwondo or wrestling as an intervention modality was found. Table 2 presents a summary of the analysed variables for each of the studies selected.

**Table 2:** Characteristics of the included studies that analyse the effects of Olympic combat sports on middle-aged and older people health-related quality of life.

**Study**	**Country**	**Study design**	**Initial health of the Sample**	**Groups**	**Activities developed in the intervention**	**Training volume**			**Training intensity**	**Measurement of HRQoL**	**Main outcomes of the studies**	**Analysis of the systematic review**
						**Total duration (weeks)**	**Frequency (weekly)**	**Time per session (min)**				
Ciaccioni et al. (2019)	Italy	RCT	Apparently healthy	40 older people (age between 64 and 77 years). EG: *n =* 19, 10 male and 9 female. CG: *n =* 21, 12 male and 9 female.	EG: judo CG: they were asked to maintain their usual activities.	16 16	2 NR	60 NR	Moderate to vigorous NR	The Health Survey Short Form (SF-12), version 2	The judo group and control group did not reveal significant effects on HRQoL.	Data analysis' effect size (ES) of judo group: Physical health: *d*= 0.48^¶^ Mental health: *d*= 0.19
Combs et al. (2013)	United States of America	RCT	Parkinson's disease	31 older people (mean age 67.3 years). EG: *n =* 17, 11 male and 6 female. CG: multi-component training (MCT); *n =* 14, 10 male and 4 female.	EG: boxing CG: MCT (strengthening exercises, endurance training, and balance activities).	12 12	2–3 2–3	90 90	NR NR	Parkinson's disease quality of life scale (PDQL)	The boxing and control groups reported a significant increase (*p* < 0.05) of the HRQoL scores without reporting differences and interactions between the groups.	Data analysis' ES of boxing group: Total score: *d* = 0.19
Dawson et al. (2020)	United States of America	Longitudinal	Parkinson's disease	47 older people (34 male and 13 female; mean age 68.3 years). Distributed in new participants (*n =* 23) and returning participants (*n =* 24).	EG: All participated in Rock Steady Boxing (RSB).	16	3	90	NR	Brief quality-of-life survey the Euroquol-5D (EQ-5D)	New participants to RSB, but not returning participants, reported a slight but statistically significant reduction in pain (*p* = 0.0437) on the EQ-5D questionnaire.	Data analysis' ES of boxing group: NR
Fleisher et al. (2020)	United States of America	NRT	Parkinson's disease	15 older people (7 male and 8 female; mean age 63.9 years).	EG: All participated in the karate program.	10	2	60	NR	The Parkinson's Disease Questionnaire-8 (PDQ-8)	HRQoL significantly improved (*p =* 0.01; ES = 0.83), passing from 25.3 to 19.3.	Data analysis' ES of karate group: Total score: *d* = 0.30^¶^
Jansen et al. (2017)	Germany	RCT	Apparently healthy	54 older people (mean age 63.5 years). EG: *n =* 23, 6 male and 17 female. MBSR group: *n =* 14, 6 male and 8 female. CG: *n =* 17, 9 male and 8 female.	EG: karate MBSR group: mindfulness-based stress reduction (MBSR). CG: they were asked to maintain their usual activities.	8 8 8	2 2 NR	60 60 NR	NR NR NR	The Health Survey Short Form (SF-12)	There was a significant main effect of time concerning the mental summary score of the SF-12 (*p =* 0.027; *n^2^* = 0.10). Yet, there was also a significant interaction effect of Group × Time (*p =* 0.05; *n^2^* = 0.12). *Post-hoc* analyses revealed that this improvement was only significant within the karate group (*p =* 0.011) but not significant in the MBSR group (*p =* 0.242), nor the control group (*p =* 0.320).	Data analysis' ES of karate group: Physical health: *d* = −0.19 Mental health: *d* = 0.72^°^
Lantheaume et al. (2017)	France	Longitudinal	Breast cancer	10 middle-aged female (mean age 47.9 years)	EG: All participated in the fencing program.	24	1	60	NR	Functional Assessment of Cancer Therapy (FACT) quality of life	There was a trend of improvement in general HRQoL scores, emotional well-being and functional well-being. Although without presenting significant changes.	Data analysis' ES of fencing group: Total score: *d* = 0.21^¶^ Emotional well-being: *d* = 0.23^¶^ Functional well-being: *d* = 0.26^¶^ Physical well-being: *d* = 0.02 Family and social well-being: *d* = 0.14
Marie-Ludivine et al. (2010)	Canada	Longitudinal	Apparently healthy	15 middle-aged males (mean age 56.7 years).	EG: All participated in the karate program.	48	3	90	NR	The French version of the 36-item Short Form Health Survey (SF-36).	Improved scores related to physical health after 6 and 12 months. Specifically, increased significantly to physical health (*p =* 0.01), physical functioning (*p =* 0.02), general health perception (*p =* 0.01), and vitality (*p* < 0.01), and a significant reduction to body pain (*p =* 0.04). However, we did not see any changes in mental health status.	Data analysis' ES of karate group: Physical health: *d* = 4.45^†^ Mental health: *d* = −0.53^°^

*CG, control group; d, effect size; EQ-5D, Brief quality-of-life survey the Euroquol-5D; EG, experimental group; ES, effect size; FACT, Functional Assessment of Cancer Therapy; Groups, total number, mean age of participants, intervention groups, and gender; HRQoL, health-related quality of life; MBSR, mindfulness-based stress reduction; MCT, multi-component training; NR, not reported; NRT, non-randomised trial; PDQ-8, The Parkinson's Disease Questionnaire-8; PDQL, Parkinson's disease quality of life scale; RCT, randomised controlled trial; RSB, Rock Steady Boxing; SF-12, The Health Survey Short Form; SF-36, Short Form Health Survey*.

¶ *small effect*;

° *moderate effect*;

† *strong effect*.

### Sample Characteristics

One study had 10 participants (Lantheaume et al., 2017), five had 15 to 50 (Marie-Ludivine et al., 2010; Combs et al., 2013; Ciaccioni et al., 2019; Dawson et al., 2020; Fleisher et al., 2020), and one had more than 50 participants (Jansen et al., 2017), which totalled a sample of 212 participants (43.4% female) with a mean age of 63.7 years. Of them, 25 had a mean age of 52.3 years (Marie-Ludivine et al., 2010; Lantheaume et al., 2017), and 187 were older than 60 years (Combs et al., 2013; Jansen et al., 2017; Ciaccioni et al., 2019; Dawson et al., 2020; Fleisher et al., 2020).

Regarding the sample's initial health level, three studies included older people with Parkinson's disease (Combs et al., 2013; Dawson et al., 2020; Fleisher et al., 2020), one study included middle-aged female with breast cancer or surgery for breast cancer (mastectomy) (Lantheaume et al., 2017), and three studies indicate that their participants were functionally independent middle-aged and older people with no apparent health problems (Marie-Ludivine et al., 2010; Jansen et al., 2017; Ciaccioni et al., 2019).

### Interventions Conducted and Dosing

Concerning the activities developed in the training protocols with OCS, for the boxing modality, Combs et al. (2013) indicate that specific activities of cardiorespiratory capacity are developed distributed as a training circuit without detailing the exercises, while Dawson et al. (2020) point out that the training circuits included endurance, resistance training, agility, and fine motor skills activities with the use of elastic bands, tennis balls and basketballs, ropes, and medicine balls, following the recommendations of the Boxing-based Fitness Programme (Rock Steady Boxing, 2011). Both studies agree that older people used gloves and punching bags without making contact with other people while boxing (Combs et al., 2013; Dawson et al., 2020). In relation to the intervention with judo (Ciaccioni et al., 2019), the sessions began with light routines and dynamic movements of the whole body imitating judo techniques, followed by specific standing, ground, passive, and active techniques performed individually and in pairs, ending with choreographies or specific forms of judo. The karate modality was used by three studies (Marie-Ludivine et al., 2010; Jansen et al., 2017; Fleisher et al., 2020). In general, the activities consisted of specific movements of the lower extremities (postures and kicks) and upper extremities (punches and blocks) and combinations of both, practised individually and in pairs, in addition to specific choreographies or forms that were adapted to the age of the people. Regarding the fencing modality (Lantheaume et al., 2017), the sessions were based on the recommendations of Hornus-Dragne et al. (2013), which included movements in front of a mirror and on targets to polish the basic fencing actions, performed individually and in pairs. In addition, as protection, female with a mastectomy had to place the opposite hand on the operated breast and took breaks when deemed appropriate (Hornus-Dragne et al., 2013; Lantheaume et al., 2017). The sessions with OCS were led mainly by certified and experienced instructors in the modalities described (Marie-Ludivine et al., 2010; Jansen et al., 2017; Lantheaume et al., 2017; Ciaccioni et al., 2019; Dawson et al., 2020; Fleisher et al., 2020) or supervised by a staff of professionals (Combs et al., 2013).

The duration of the interventions varied: five took place between 8 and 16 weeks (Combs et al., 2013; Jansen et al., 2017; Ciaccioni et al., 2019; Dawson et al., 2020; Fleisher et al., 2020), one lasted 24 weeks (Lantheaume et al., 2017), and one lasted 48 weeks (Marie-Ludivine et al., 2010). The frequency of training ranged between one and three weekly sessions, with a duration per session of 60 (Jansen et al., 2017; Lantheaume et al., 2017; Ciaccioni et al., 2019; Fleisher et al., 2020) or 90 min (Marie-Ludivine et al., 2010; Combs et al., 2013; Dawson et al., 2020). Training intensity was reported only by Ciaccioni et al. (2019), which remained moderate to vigorous.

### Measurement of Health-Related Quality of Life

The selected studies used different instruments to assess the effects of the OCS interventions on HRQoL. One study used the Health Survey Short Form (SF-36) (Marie-Ludivine et al., 2010), with the validated version in French (Leplège, 2001), an instrument made up of 36 items that assess different dimensions of health. Two studies measured with the short version of the Health Survey Short Form (SF-12), version 1 (Jansen et al., 2017) and version 2 (Ciaccioni et al., 2019), which is composed of 12 items (Jenkinson et al., 1997). Both surveys (SF-36 and SF-12) measure attributes of eight health dimensions (physical function, physical role, body pain, general health, vitality, social function, emotional role, and mental health), which can be grouped into two settings of health (physical health and mental health). Here, the values range from 0 to 100 points (Jenkinson et al., 1997; Leplège, 2001), and where a higher value means better HRQoL. On the other hand, Combs et al. (2013) used the Parkinson's Disease Quality of Life scale (PDQL), an instrument that has 37 items with values ranging between 37 and 185 points, where a higher value indicates a better HRQoL (De Boer et al., 1996). For their part, Fleisher et al. (2020) used the short form 8-item Parkinson's Disease Questionnaire (PDQ-8), which measures the quality of life in a concise and specific way. The PDQ-8 provides a range that goes from 0 to 100 points, where a higher value means worse HRQoL (Jenkinson and Fitzpatrick, 2007). Dawson et al. (2020) evaluated with the Brief Quality-of-life Survey the Euroquol-5D (EQ-5D), an instrument composed of five questions on mobility, self-care, pain, habitual activities, and psychological state, with values ranging from 0 to 100 points (Brooks, 1996; Schrag et al., 2000), where a higher value means better HRQoL. Finally, one study used the Functional Assessment of Cancer Therapy (FACT) quality of life (Lantheaume et al., 2017). This instrument includes four health dimensions: physical well-being, family and social well-being, emotional well-being, and functional well-being. It comprises 37 items with a value ranging from 0 to 148 points, where a higher score means better HRQoL (Bonomi et al., 1996).

### Main Outcomes in Health-Related Quality of Life

The selected studies did not qualify to meta-analyse their results. However, six studies provided data to determine the ES of HRQoL in the intervention groups treated with OCS (Marie-Ludivine et al., 2010; Combs et al., 2013; Jansen et al., 2017; Lantheaume et al., 2017; Ciaccioni et al., 2019; Fleisher et al., 2020). Regarding the total score, three studies provided data through various instruments (i.e., PDQL, PDQ-8, FACT), reporting beneficial changes with a small ES (*d* < 0.40) on HRQoL in the intervention groups with boxing and karate in older people with Parkinson's disease (Combs et al., 2013; Fleisher et al., 2020) and in middle-aged female with breast cancer who participated in fencing (Lantheaume et al., 2017).

Regarding the health settings measured through the SF-12 and SF-36, three studies provided data, reporting beneficial changes on physical health with a small ES in older people (*d* = 0.49) (Ciaccioni et al., 2019) and strong ES (*d* = 4.45) in middle-aged males (Marie-Ludivine et al., 2010) without apparent health problems and intervened with judo and karate, respectively. While mental health reported contradictory results, on the one hand, there was a negative change with a moderate ES (*d* = −0.53) in middle-aged males (Marie-Ludivine et al., 2010), and, on the other hand, a beneficial change where reported with a moderate ES (*d* = 0.72) in older people (Jansen et al., 2017) intervened with karate. Regarding the health dimensions, a study provided data through the FACT (Lantheaume et al., 2017), reporting beneficial changes with a small ES for emotional (*d* = 0.23) and functional (*d* = 0.26) well-being in middle-aged female who participated in fencing and had breast cancer.

On the other hand, when analysing the results reported by the selected studies, it is noted that five studies report beneficial changes on HRQoL in older people that intervened with boxing (Combs et al., 2013; Dawson et al., 2020) and middle-aged and older people intervened with karate (Marie-Ludivine et al., 2010; Jansen et al., 2017; Fleisher et al., 2020). Specifically, Combs et al. (2013) reported a significant increase in the total PDQL score in the boxing group (*p* = 0.012) and the control group (with multi-component training, *p* = 0.022). Dawson et al. (2020) only reported a slight but significant reduction in pain (*p* = 0.0437) in new participants to the boxing intervention, as measured by the EQ-5D. In karate, Fleisher et al. (2020) reported a significant reduction (*p* = 0.01) in the total score of the PDQ-8 in older people, which indicates an improvement in the HRQoL. Jansen et al. (2017) reported a significant improvement (*p* = 0.011) only in karate group (with older people) for mental health measured through the SF-12. For their part, Marie-Ludivine et al. (2010) point out a significant increase in physical health (*p* = 0.01), physical functioning (*p* = 0.02), perception of general health (*p* = 0.01), and vitality (*p* < 0.01) and a significant reduction in body pain (*p* = 0.04) in the middle-aged males measured with the SF-36, but without changes in the mental health setting. Despite an improving trend in the physical and mental health of SF-12 in the older people that intervened with judo, no significant changes were evidenced (Ciaccioni et al., 2019). A similar situation was reflected in middle-aged female who participated in the training with fencing, demonstrating an improvement trend for physical, family and social, emotional, and functional well-being and total FACT score, but without reporting significant changes (Lantheaume et al., 2017).

### Adherence and Attrition

Another relevant aspect corresponds to the retention or adherence of the participants in OCS interventions. One study reported no attrition of its participants for fencing workouts (Lantheaume et al., 2017). Five studies achieved adherence between 71 and 96% for interventions with boxing (Dawson et al., 2020), judo (Ciaccioni et al., 2019), and karate (Marie-Ludivine et al., 2010; Jansen et al., 2017; Fleisher et al., 2020). Meanwhile, a study with boxing reports retention of 64% (Combs et al., 2013). The main reasons for attrition were related to diseases or personal problems (Marie-Ludivine et al., 2010; Combs et al., 2013; Fleisher et al., 2020), not complying with the minimum attendance at training sessions (Ciaccioni et al., 2019; Dawson et al., 2020; Fleisher et al., 2020), loss of interest (Marie-Ludivine et al., 2010; Combs et al., 2013), and conflicts with the training schedule (Combs et al., 2013; Fleisher et al., 2020). Only one study did not indicate the reasons for the attrition of its participants (Jansen et al., 2017).

## Discussion

The present systematic review aimed to analyse the effects of OCS interventions on HRQoL in adults aged 45 and older. After reviewing 1,151 records, seven studies met the inclusion criteria and scored 60% or more (moderate-high quality) of the established score for methodological quality. The main result of our review indicates that interventions based on OCS produce beneficial changes and an improvement trend with a small and moderate ES in HRQoL in both male and female, healthy participants, participants with Parkinson's disease, and participants with breast cancer. This result reinforces the scientific literature that has reported a positive impact of martial arts and combat sports on the health status in different population groups (Bu et al., 2010; Origua Rios et al., 2018; Moore et al., 2020; Valdés-Badilla et al., 2021).

Six of the studies analysed in our systematic review reported beneficial effects with a small and moderate ES on the total score, settings, or health dimensions of HRQoL in the intervention groups with OCS (Marie-Ludivine et al., 2010; Combs et al., 2013; Jansen et al., 2017; Lantheaume et al., 2017; Ciaccioni et al., 2019; Fleisher et al., 2020). It has been reported that the regular practise of PA promotes greater physical self-concept and satisfaction with life, which has a positive impact on mood and the quality of life perception (Arruza et al., 2008). Likewise, maintaining or improving HRQoL favours self-esteem and reduces the negative perception of ageing (Ingrand et al., 2018), which positively impacts general well-being (Marquez et al., 2020).

The changes observed for HRQoL, according to the initial health level of the participants in the interventions based on OCS, it can be indicated that older people with Parkinson's disease achieved beneficial changes with a small ES on the total score of the HRQoL after 10 (Fleisher et al., 2020) or 12 weeks (Combs et al., 2013) of karate and boxing. One study reports a significant decrease in pain (Dawson et al., 2020) after 16 weeks of boxing in older people without providing data to obtain the ES. Parkinson's is a progressive nervous system disease that affects body movement, causing muscle stiffness, bradykinesia, and tremor at rest (Sorrell, 2017). Various researches have used PA strategies to attenuate its effects; for example, a recent meta-analysis reported that studies that used resistance training interventions had not shown superiority over other PA strategies to support their use as a rehabilitation technique for this disease (Saltychev et al., 2016). On the other hand, our systematic review found only one study that used OCS, specifically fencing, for 24 weeks in middle-aged female with breast cancer, reporting beneficial changes with a small ES in the total score and emotional and functional well-being (Lantheaume et al., 2017). Breast cancer is a type of cancer that forms in breast cells, more common in female (Strunk et al., 2018). It has been suggested that PA interventions positively impact the HRQoL of people with different types of cancer (Mishra et al., 2012); in particular, female with breast cancer increase their self-esteem, body image, and emotional well-being. Despite the promising results of our HRQoL systematic review, further research is required to propose OCS as treatment alternatives for Parkinson's disease and breast cancer.

Regarding the changes found in the HRQoL of middle-aged and older people without apparent health problems who participated in OCS interventions, improvements in physical health are reported with a small ES (Ciaccioni et al., 2019) and strong ES (Marie-Ludivine et al., 2010), while mental health achieved contradictory results, reporting negative changes with a moderate ES in middle-aged male (Marie-Ludivine et al., 2010) and favourable changes with a moderate ES in older people (Jansen et al., 2017). In this context, a previous systematic review (Moore et al., 2020) reported that the groups that practise martial arts regularly present a positive and significant effect with a small ES on well-being (*d* = 0.346) and moderate ES on internalisation of mental health (*d* = 0.620) with respect to control groups. The regular practise of OCS can achieve beneficial changes in the physical health of participants considered healthy; however, more study is still required to determine the effects at the mental health level.

Concerning the dosage used for the interventions based on OCS, it can be indicated that they lasted between 8 and 48 weeks, distributed in one to three weekly sessions with a time per session of 60–90 min (Marie-Ludivine et al., 2010; Combs et al., 2013; Jansen et al., 2017; Lantheaume et al., 2017; Ciaccioni et al., 2019; Dawson et al., 2020; Fleisher et al., 2020). The intensity was only reported by one study (Ciaccioni et al., 2019), which kept it from moderate to vigorous. International PA recommendations suggest performing between 150 and 300 min of moderate-intensity PA or 75–150 min of vigorous-intensity PA per week (Bull et al., 2020), including at least two strengthening sessions focused on the large muscle groups (Fragala et al., 2019). Although our systematic review does not provide conclusive information, it is possible to note that the dosage used for interventions based on OCS is aligned with current PA recommendations for middle-aged and older people (Fragala et al., 2019; Bull et al., 2020). It also shows that it is possible to adapt OCS activities to people with different initial health levels, increasing their HRQoL, suggesting their use as PA alternatives for adults aged 45 and older.

Regarding the adherence achieved by the participants in the interventions based on OCS, a mean adherence rate >80% was reported. In addition, the leading causes of attrition are related to diseases, personal problems, not meeting minimum attendance, or conflicts with the training schedule, while only two studies reported a loss of interest (Marie-Ludivine et al., 2010; Combs et al., 2013). This is relevant since PA workshops based on multi-component training and healthy dance in older people implemented by a government institution reported adherence of 54.6% after 16 weeks of participation (Valdés-Badilla et al., 2020). OCS may be PA strategies with high adherence (average >80%), which could transform them into an opportunity to achieve greater outreach and participation in middle-aged and older people, without postponing the benefits for their health (Bu et al., 2010; Origua Rios et al., 2018; Valdés-Badilla et al., 2021).

The main strengths of this review are as follows: (i) the methods used for the selection and assessments of studies followed the recommendations of PRISMA, PROSPERO protocols, and the Downs and Black's checklist; (ii) five generic databases (SCOPUS, PubMed/MEDLINE, Web of Science, PsycINFO, EBSCO) were used for information retrieval, increasing precision and reducing possible bias in the results obtained; and (iii) was not limitations on languages or publication date of the studies selection, which widened the search scope. As limitations, the following can be pointed out: (i) the diversity of instruments used to measure HRQoL and the small number of randomised-controlled trials that made a meta-analysis of the data impossible, due to requiring three or more studies to analyse their results (Valentine et al., 2010), and (ii) not finding studies that used taekwondo or wrestling modality as an intervention, and the diversity in the sample's initial health level, which reduces the generalisation of the data.

Even though OCS are widely disseminated modalities worldwide, their use as PA strategies for adults aged 45 and older seems rare (Bu et al., 2010; Valdés-Badilla et al., 2021). In this regard, and considering the studies discussed in this systematic review, interventions based on OCS for middle-aged and older people could be safe, with high adherence and positively impact on HRQoL. However, our results should be considered with caution due to the heterogeneity of the participants in OCS interventions, which does not allow for definitive recommendations on dosage (e.g., frequency, volume, intensity, density), technical foundations (e.g., postures, displacements, blows, projections, kicks) and modality (boxing, judo, karate, fencing), according to the range of age and initial health level of the participants. Therefore, more studies with high methodological quality (e.g., double-blind randomisation, supervised control groups, previously register their research protocols) and more description of the physical exercise (technical foundations) are needed to dose and select the most appropriate activities and modality for middle-aged and older people.

## Conclusion

Interventions based on OCS produce beneficial effects with a small and moderate ES on HRQoL in male and female aged 45 and older who are healthy participants, participants with Parkinson's disease, and participants with breast cancer. OCS are PA strategies that can be adapted to different population groups, considering the characteristics of the people and their initial health level.

## Data Availability Statement

The original contributions presented in the study are included in the article/Supplementary Material, further inquiries can be directed to the corresponding author/s.

## Author Contributions

PV-B, TH-V, and EG-M wrote the first draft of the manuscript, collected data, and analysed and interpreted the data. PD-F, CN-E, MM-A, and DA revised the original manuscript. All authors read and approved the final manuscript.

## Conflict of Interest

The authors declare that the research was conducted in the absence of any commercial or financial relationships that could be construed as a potential conflict of interest.

## Publisher's Note

All claims expressed in this article are solely those of the authors and do not necessarily represent those of their affiliated organizations, or those of the publisher, the editors and the reviewers. Any product that may be evaluated in this article, or claim that may be made by its manufacturer, is not guaranteed or endorsed by the publisher.

## References

[B1] Arruza J. A. Arribas S. Gil De Montes L. Irazusta S. Romero S. Cecchini J. A. (2008). The impact of duration in sport and physical activity on the psychological well-being. Rev. Internacional de Medicina y Ciencias de la Actividad Física y el Deporte 8, 171–183. 31399142

[B2] Bonomi A. E. Cella D. F. Hahn E. A. Bjordal K. Sperner-Unterweger B. Gangeri L. . (1996). Multilingual translation of the Functional Assessment of Cancer Therapy (FACT) quality of life measurement system. Qual. Life Res. 5, 309–320. 10.1007/BF00433915 8763799

[B3] Bromley S. J. Drew M. K. Talpey S. McIntosh A. S. Finch C. F. (2018). A systematic review of prospective epidemiological research into injury and illness in Olympic combat sport. Br. J. Sports. Med. 52, 8–16. 10.1136/bjsports-2016-097313 28954799

[B4] Brooks R. (1996). EuroQol: the current state of play. Health Policy 37, 53–72. 10.1016/0168-8510(96)00822-6 10158943

[B5] Bu B. Haijun H. Yong L. Chaohui Z. Xiaoyuan Y. Singh M. F. (2010). Effects of martial arts on health status: a systematic review. J. Evid. Based Med. 3, 205–219. 10.1111/j.1756-5391.2010.01107.x 21349072

[B6] Bull F. C. Al-Ansari S. S. Biddle S. Borodulin K. Buman M. P. Cardon G. . (2020). World Health Organization 2020 guidelines on physical activity and sedentary behaviour. Br. J. Sports. Med. 54, 1451–1462. 10.1136/bjsports-2020-102955 33239350 PMC7719906

[B7] Cadore E. L. Rodríguez-Mañas L. Sinclair A. Izquierdo M. (2013). Effects of different exercise interventions on risk of falls, gait ability, and balance in physically frail older people: a systematic review. Rejuvenation Res. 16, 105–114. 10.1089/rej.2012.1397 23327448 PMC3634155

[B8] Ciaccioni S. Capranica L. Forte R. Chaabene H. Pesce C. Condello G. (2019). Effects of a judo training on functional fitness, anthropometric, and psychological variables in old novice practitioners. J. Aging Phys. Act. 27, 831–842. 10.1123/japa.2018-0341 31034297

[B9] Cohen J. (1992). Quantitative methods in psychology: a power primer. Psychol. Bull. 112, 1155–1159. 10.1037/0033-2909.112.1.155

[B10] Combs S. A. Diehl M. D. Chrzastowski C. Didrick N. McCoin B. Mox N. . (2013). Community-based group exercise for persons with Parkinson disease: a randomised controlled trial. NeuroRehabilitation 32, 117–124. 10.3233/NRE-130828 23422464

[B11] Cunningham C. O'Sullivan R. Caserotti P. Tully M. A. (2020). Consequences of physical inactivity in older people: a systematic review of reviews and meta-analyses. Scand. J. Med. Sci. Sports 30, 816–827. 10.1111/sms.13616 32020713

[B12] Dawson R. A. Sayadi J. Kapust L. Anderson L. Lee S. Latulippe A. . (2020). Boxing exercises as therapy for parkinson disease. Top. Geriatr. Rehabil. 36, 160–165. 10.1097/TGR.0000000000000275

[B13] De Boer A. G. Wijker W. Speelman J. D. De Haes J. C. (1996). Quality of life in patients with Parkinson's disease: development of a questionnaire. J. Neurol. Neurosurg. Psychiatry 61, 70–74. 10.1136/jnnp.61.1.70 8676165 PMC486462

[B14] Downs S. H. Black N. (1998). The feasibility of creating a checklist for the assessment of the methodological quality both of randomised and non-randomised studies of health care interventions. J. Epidemiol. Commun. Health 52, 377–384. 10.1136/jech.52.6.377 9764259 PMC1756728

[B15] Fleisher J. E. Sennott B. J. Myrick E. Niemet C. J. Lee M. Whitelock C. M. . (2020). KICK out PD: feasibility and quality of life in the pilot karate intervention to change kinematic outcomes in Parkinson's Disease. PLoS ONE 15:e0237777. 10.1371/journal.pone.0237777 32903267 PMC7480843

[B16] Fragala M. S. Cadore E. L. Dorgo S. Izquierdo M. Kraemer W. J. Peterson M. D. . (2019). Resistance training for older people: position statement from the national strength and conditioning association. J. Strength Condition. Res. 33, 2019–2052. 10.1519/JSC.0000000000003230 31343601

[B17] Freke M. Kemp J. L. Svege I. Risberg M. A. Semciw A. I. Crossley K. M. (2016). Physical impairments in symptomatic femoroacetabular impingement: a systematic review of the evidence. Br. J. Sports. Med. 50, 1180–1180. 10.1136/bjsports-2016-096152 27301577

[B18] Giuliano C. Karahalios A. Neil C. Allen J. Levinger I. (2017). The effects of resistance training on muscle strength, quality of life and aerobic capacity in patients with chronic heart failure—a meta-analysis. Int. J. Cardiol. 227, 413–423. 10.1016/j.ijcard.2016.11.023 27843045

[B19] Grande G. D. Oliveira C. B. Morelhão P. K. Sherrington C. Tiedemann A. Pinto R. Z. . (2020). Interventions promoting physical activity among older people: a systematic review and meta-analysis. Gerontologist 60, e583–e599. 10.1093/geront/gnz167 31868213

[B20] Grgic J. Schoenfeld B. J. Davies T. B. Lazinica B. Krieger J. W. Pedisic Z. (2018). Effect of resistance training frequency on gains in muscular strength: a systematic review and meta-analysis. Sports Med. 48, 1207–1220. 10.1007/s40279-018-0872-x 29470825

[B21] Hornus-Dragne D. Manenc J. Farnarier J. Cuvier C. Lucas C. Riviére D. (2013). L'Escrime chez les femmes opérées d'un cancer du sein. Paris. Available online at: https://solutionriposte.monsite-orange.fr/file/b8ff35a037a31f73460cd7b83ea86365.pdf

[B22] Ingrand I. Paccalin M. Liuu E. Gil R. Ingrand P. (2018). Positive perception of aging is a key predictor of quality-of-life in aging people. PLoS ONE 13:e0204044. 10.1371/journal.pone.0204044 30281672 PMC6169874

[B23] Jansen P. Dahmen-Zimmer K. Kudielka B. M. Schulz A. (2017). Effects of karate training versus mindfulness training on emotional well-being and cognitive performance in later life. Res. Aging 39, 1118–1144. 10.1177/0164027516669987 27688143

[B24] Jenkinson C. Fitzpatrick R. (2007). Cross-cultural evaluation of the short form 8-item Parkinson's Disease Questionnaire (PDQ-8): results from America, Canada, Japan, Italy and Spain. Parkinsonism Relat. Disord. 13, 22–28. 10.1016/j.parkreldis.2006.06.006 16931104

[B25] Jenkinson C. Layte R. Jenkinson D. Lawrence K. Petersen S. Paice C. . (1997). A shorter form health survey: can the SF-12 replicate results from the SF-36 in longitudinal studies? J. Public Health 19, 179–186. 10.1093/oxfordjournals.pubmed.a024606 9243433

[B26] Kojima G. Iliffe S. Jivraj S. Walters K. (2016). Association between frailty and quality of life among community-dwelling older people: a systematic review and meta-analysis. J. Epidemiol. Commun. Health 70, 716–721. 10.1136/jech-2015-206717 26783304

[B27] Lantheaume S. Fabre F. Fisch C. Motak L. Massol P. Lantheaume S. . (2017). Breast cancer, adapted physical activity and quality of life. Ann. Med. Psychol. 175, 841–848. 10.1016/j.amp.2016.03.016

[B28] Leplège A. (2001). Le questionnaire MOS SF-36: Manuel de l'utilisateur et guide d'interprétation des scores: De Boeck Secundair. Available online at: https://bdsp-ehesp.inist.fr/vibad/index.php?action=~getRecordDetail&idt=236340 (accessed June 17, 2021).

[B29] Livingston G. Huntley J. Sommerlad A. Ames D. Ballard C. Banerjee S. . (2020). Dementia prevention, intervention, and care: 2020 report of the Lancet Commission. Lancet 396, 413–446. 10.1016/S0140-6736(20)30367-6 32738937 PMC7392084

[B30] Marie-Ludivine C. Papouin G. Saint-Val P. Lopez A. (2010). Effect of adapted karate training on quality of life and body balance in 50-year-old men. Open Access J. Sports Med. 1:143. 10.2147/OAJSM.S12479 24198552 PMC3781864

[B31] Marquez D. X. Aguiñaga S. Vásquez P. M. Conroy D. E. Erickson K. I. Hillman C. . (2020). A systematic review of physical activity and quality of life and well-being. Transl. Behav. Med. 10, 1098–1109. 10.1093/tbm/ibz198 33044541 PMC7752999

[B32] Mendonça D. L. Alonso A. C. Greve J. M. Garcez-Leme L. E. (2017). Assessment of the quality of life, muscle strength, and dynamic balance of elderly Kendo players. Clinics 72, 661–666. 10.6061/clinics/2017(11)03 29236911 PMC5706064

[B33] Mishra S. I. Scherer R. W. Geigle P. M. Berlanstein D. R. Topaloglu O. Gotay C. C. . (2012). Exercise interventions on health-related quality of life for cancer survivors. Cochrane Database Syst. Rev. 8:CD007566. 10.1002/14651858.CD007566.pub2 22895961 PMC7387117

[B34] Moore B. Dudley D. Woodcock S. (2020). The effect of martial arts training on mental health outcomes: a systematic review and meta-analysis. J. Bodyw. Mov. Ther. 24, 402–412. 10.1016/j.jbmt.2020.06.017 33218541

[B35] Origua Rios S. Marks J. Estevan I. Barnett L. M. (2018). Health benefits of hard martial arts in adults: a systematic review. J. Sports Sci. 36, 1614–1622. 10.1080/02640414.2017.1406297 29157151

[B36] Page M. J. McKenzie J. E. Bossuyt P. M. Boutron I. Hoffmann T. C. Mulrow C. D. . (2021). The PRISMA 2020 statement: an updated guideline for reporting systematic reviews. Br. Med. J. 372:n71. 10.1136/bmj.n71 33782057 PMC8005924

[B37] Reis R. S. Salvo D. Ogilvie D. Lambert E. V. Goenka S. Brownson R. C. . (2016). Scaling up physical activity interventions worldwide: stepping up to larger and smarter approaches to get people moving. Lancet 388, 1337–1348. 10.1016/S0140-6736(16)30728-0 27475273 PMC5193005

[B38] Rock Steady Boxing (2011). Fighting Back Against Parkinson's. Available online at: https://www.rocksteadyboxing.org/about/ (accessed May 19, 2021)

[B39] Saltychev M. Bärlund E. Paltamaa J. Katajapuu N. Laimi K. (2016). Progressive resistance training in Parkinson's disease: a systematic review and meta-analysis. BMJ Open 6:e008756. 10.1136/bmjopen-2015-008756 26743698 PMC4716165

[B40] Schrag A. Selai C. Jahanshahi M. Quinn N. P. (2000). The EQ-5D—a generic quality of life measure—is a useful instrument to measure quality of life in patients with Parkinson's disease. J. Neurol. Neurosurg. Psychiatry 69, 67–73. 10.1136/jnnp.69.1.67 10864606 PMC1737007

[B41] Song R. Grabowska W. Park M. Osypiuk K. Vergara-Diaz G. P. Bonato P. . (2017). The impact of Tai Chi and Qigong mind-body exercises on motor and non-motor function and quality of life in Parkinson's disease: a systematic review and meta-analysis. Parkinsonism Relat. Disord. 41, 3–13. 10.1016/j.parkreldis.2017.05.019 28602515 PMC5618798

[B42] Sorrell J. M. (2017). Living with Parkinson's disease: staying determined. J. Psychosoc. Nurs. Mental Health Serv. 55, 15–18. 10.3928/02793695-20170330-03 28407154

[B43] Strunk M. A. Zopf E. M. Steck J. Hamacher S. Hallek M. Baumann F. T. (2018). Effects of KYUSHO JITSU on physical activity-levels and quality of life in breast cancer patients. In vivo 32, 819–824. 10.21873/invivo.11313 29936464 PMC6117768

[B44] Valdés-Badilla P. Gutiérrez-García C. Pérez-Gutiérrez M. Vargas-Vitoria R. López-Fuenzalida A. (2019). Effects of physical activity governmental programs on health status in independent older people: a systematic review. J. Aging Phys. Act. 27, 265–275. 10.1123/japa.2017-0396 29989461

[B45] Valdés-Badilla P. Guzmán-Muñoz E. Ramírez-Campillo R. Godoy-Cumillaf A. Concha-Cisternas Y. Ortega-Spuler J. . (2020). Changes in anthropometric parameters and physical fitness in older people after participating in a 16-week physical activity program. Revista de la Facultad de Medicina 68, 375–382. 10.15446/revfacmed.v68n3.75817

[B46] Valdés-Badilla P. Herrera-Valenzuela T. Ramirez-Campillo R. Aedo-Muñoz E. Báez-San Martín E. Ojeda-Aravena A. . (2021). Effects of olympic combat sports on older people' health status: a systematic review. Int. J. Environ. Res. Public Health 18:7381. 10.3390/ijerph18147381 34299833 PMC8303637

[B47] Valentine J. C. Pigott T. D. Rothstein H. R. (2010). How many studies do you need? A primer on statistical power for meta-analysis. J. Educ. Behav. Statist. 35, 215–247. 10.3102/1076998609346961

[B48] Vargas-Vitoria R. Larena J. Rodríguez M. Arellano R. Valdés-Badilla P. (2021). Efectos de un programa multicomponente sobre medidas antropométricas, condición física y calidad de vida relacionada con la salud en personas mayores. Nutrición Clínica y Dietética Hospitalaria 41, 69–75. 10.12873/411vargas

[B49] Warburton D. E. R. Bredin S. S. D. (2017). Health benefits of physical activity: a systematic review of current systematic reviews. Curr. Opin. Cardiol. 32, 541–556. 10.1097/HCO.0000000000000437 28708630

[B50] Zubala A. MacGillivray S. Frost H. Kroll T. Skelton D. A. Gavine A. . (2017). Promotion of physical activity interventions for community dwelling older people: a systematic review of reviews. PLoS ONE 12:e0180902. 10.1371/journal.pone.0180902 28700754 PMC5507305

