# Supplementary material for: Effects of Olympic Combat Sports on Health-Related Quality of Life in Middle-Aged and Older People: A Systematic Review
Source: Front Psychol. 2022 Jan 5;12:797537. doi: 10.3389/fpsyg.2021.797537 (PMC8769282; doi:10.3389/fpsyg.2021.797537)
Supplement: Supplementary file 1 [file Data_Sheet_1.pdf]

Supplementary material

Assesment the methodological quality criteria according to the Downs and Black's (1998) checklist:

1. Is the hypothesis/aim/objective of the study clearly described?

2. Are the main outcomes to be measured clearly described in the Introduction or Methods section?

3. Are the characteristics of the patients included in the study clearly described?

4. Are the interventions of interest clearly described?

5. Are the distributions of principal confounders in each group of subjects to be compared clearly described?

6. Are the main findings of the study clearly described?

7. Does the study provide estimates of the random variability in the data for the main outcomes?

8. Have all important adverse events that may be a consequence of the intervention been reported?

9. Have the characteristics of patients lost to follow-up been described?

10. Have actual probability values been reported (e.g. 0.035 rather than <0.05) for the main outcomes except where the probability value is less than 0.001?

11. Were the subjects asked to participate in the study representative of the entire population from which they were recruited?

12. Were those subjects who were prepared to participate representative of the entire population from which they were recruited?

13. Were the staff, places, and facilities where the patients were treated representative of the treatment the majority of patients receive?

14. Was an attempt made to blind study subjects to the intervention they have received? (We did not use the responses to this question to rate the Risk of Bias given the nature of the interventions).

15. Was an attempt made to blind those measuring the weight outcomes of the intervention? (This item must have been answered Yes for a trial to have low Risk of Bias).

16. If any of the results of the study were based on "data dredging", was this made clear?

17. In trials and cohort studies, do the analyses adjust for different lengths of follow-up of patients, or in case-control studies, is the time period between the intervention and outcome the same for cases and controls?

18. Were the statistical tests used to assess the main outcomes appropriate?

19. Was compliance with the intervention/s reliable?

20. Were the main outcome measures used accurate (valid and reliable)?

21. Were the patients in different intervention groups (trials and cohort studies) or were the cases and controls (case-control studies) recruited from the same population?

22. Were study subjects in different intervention groups (trials and cohort studies) or were the cases and controls (case-control studies) recruited over the same period of time?

23. Were study subjects randomized to intervention groups?

24. Was the randomized intervention assignment concealed from both patients and health care staff until recruitment was complete and irrevocable?

25. Was there adequate adjustment for confounding in the analyses from which the main findings were drawn?

26. Were losses of patients to follow-up taken into account?

27. Did they report a power calculation?
